# Supplementary material for: Burden is in the eye of the beholder: Sensitivity of yellow fever disease burden estimates to modeling assumptions
Source: Sci Adv. 2021 Oct 13;7(42):eabg5033. doi: 10.1126/sciadv.abg5033 (PMC11559552; doi:10.1126/sciadv.abg5033)
Supplement: Supplementary file 1 — Supplementary Text Figs. S1 to S10 Table S1 References [file sciadv.abg5033_sm.pdf]

Science Advances
AAAS

Supplementary Materials for

Burden is in the eye of the beholder: Sensitivity of yellow fever disease burden estimates to modeling assumptions

T. Alex Perkins*, John H. Huber, Quan M. Tran, Rachel J. Oidtman, Magdalene K. Walters, Amir S. Siraj, Sean M. Moore

*Corresponding author. Email: taperkins@nd.edu

Published 13 October 2021, *Sci. Adv.* **7**, eabg5033 (2021)
DOI: 10.1126/sciadv.abg5033

This PDF file includes:

Supplementary Text
Figs. S1 to S10
Table S1
References

Supplementary Text

Probabilities of different infection outcomes

There were two steps in our analysis for which we needed to make assumptions about the probabilities of different infection outcomes. In Step 2, we placed priors on the proportion of reported events that were cases or deaths. In the final step of translating ensemble projections of force of infection into deaths, we made an assumption about the proportion of infections that result in death.

We based both of these assumptions on a re-analysis of data compiled by Johansson et al. (*3*). While we were amenable to using estimates of the probabilities of different infection outcomes by Johansson et al., information was only presented in that paper about marginal distributions of the probability of each infection outcome. We felt that it was important to make use of estimates for which correlation structure among the probabilities of different infection outcomes was accounted for.

A natural distribution for representing uncertainty in multiple probabilities that together sum to one is a Dirichlet distribution (*70*). Accordingly, we used maximum likelihood to estimate parameters of a Dirichlet distribution describing the probabilities of the same four infection outcomes considered by Johansson et al.: A = asymptomatic infection; M = mild symptomatic infection; S = severe symptomatic infection; and F = infection resulting in a fatality. We used all data presented in Tables 1 and 2 of Johansson et al. in which two or more combinations of infection outcomes were reported in the same row. In some cases, this included sums of infection outcomes; namely, A+M and M+S, given ambiguity in some studies about these infection outcomes. To do this, we leveraged the property that the four concentration parameters of the Dirichlet distribution—α_A, α_M, α_S, and α_F—can be summed to obtain concentration parameters of a lower-dimensional Dirichlet distribution (including a beta distribution in the case of two outcomes) that are consistent with the higher-dimensional Dirichlet distribution (*70*).

Our analysis resulted in maximum-likelihood estimates (MLE) of $\alpha_A = 8.69$, $\alpha_M = 2.74$, $\alpha_S = 4.10$, and $\alpha_F = 2.05$. This corresponds to mean estimates (and 95% credible intervals, by which we mean the 0.025-0.975 quantile range under the MLE Dirichlet parameters) of the probability that an infection results in outcomes of A, M, and S+F of 0.49 (0.27-0.72), 0.16 (0.03-0.35), and 0.35 (0.15-0.58), respectively. This compares with estimates by Johansson et al. (*3*) 0.55 (0.37-0.74), 0.33 (0.13-0.52), and 0.12 (0.05-0.26), respectively. For the probability of death given severe disease, we obtained a mean estimate (and 95% credible interval) of 0.33 (0.04-0.57), as compared to an estimate of 0.47 (0.31-0.62) by Johansson et al. Together, this meant that our mean estimate of the probability of death upon infection of 0.12 was twice that of Johansson et al. (0.05), although there was also wider uncertainty in our estimate (0.01-0.27) than that of Johansson et al. (0.02-0.12). The differences between our estimates could be due either to the different distributions we used (Dirichlet vs. binomials) or the additional step in the analysis by Johansson et al. that estimated the prevalence of infection in the outbreaks from which the underlying data came.

Variance partitioning

Given our interest in evaluating the sensitivity of disease burden and vaccination impact to model assumptions, quantifying the proportion of variance in those measures attributable to different sources was an important focus of our results. To do that, we applied the law of total variance (*71*) to draws of outputs of interest—force of infection (FOI), deaths averted, and deaths—from their posterior distributions.

For a given output of interest (e.g., force of infection), Y, and a single explanatory factor (e.g., first-level administrative unit, adm1), X, total variance in Y can be partitioned according to

$$\mathrm{Var}(Y) = \mathrm{E}[\,\mathrm{Var}(Y \mid X)\,] + \mathrm{Var}(\,\mathrm{E}[Y \mid X]\,),$$

where the first term on the right-hand side represents variance in Y not accounted for by X (which we referred to as statistical uncertainty) and the second term represents variance in Y that is accounted for by X. This equation was used to calculate the proportion of variance in $\log_{10}$ FOI (Y) attributable to adm1 (X) in Step 3 and regression model (X) in Step 4.

In the case of two explanatory factors, X_1 and X_2, the above equation can be extended to

$$\mathrm{Var}(Y) = \mathrm{E}[\,\mathrm{Var}(Y \mid X_1, X_2)\,] + \mathrm{E}[\,\mathrm{Var}(\,\mathrm{E}[Y \mid X_1, X_2] \mid X_1)\,] + \mathrm{Var}(\,\mathrm{E}[Y \mid X_1]\,),$$

where the first term on the right-hand side represents variance in Y not accounted for by X_1 or X_2, the third term represents variance in Y accounted for by X_1, and the second represents variance in Y accounted for by X_2 conditional on X_1 (*72*). This equation was used to calculate the proportion of variance in $\log_{10}$ FOI (Y), deaths averted (Y), and deaths (Y) attributable to adm1 (X_1) and serology scenario (X_2). We selected adm1 as X_1 given that spatial heterogeneity in yellow fever due to heterogeneity in underlying environmental drivers is considerable, making it logical to consider it as a primary source of variation in yellow fever's burden.

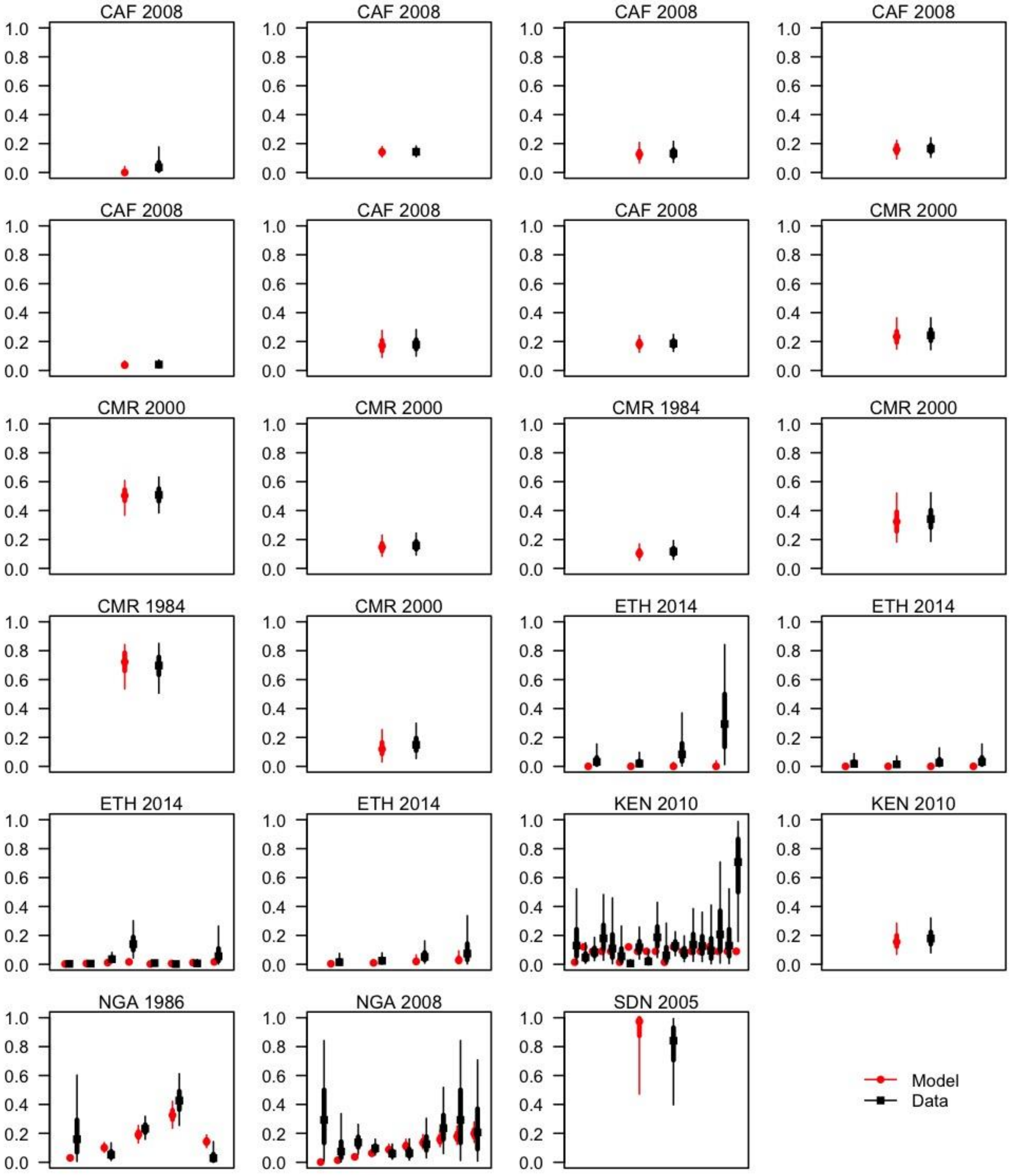

Figure S1. Comparison of seroprevalence based on estimates of force of infection from Step 1 (red) and simple estimates of seropositivity based on conjugate prior relationships (black) under serology scenario 1. The conjugate prior estimates assumed a flat beta prior with shape parameters equal to 1 and a binomial likelihood applied independently to each data point (*66*). Within each panel, different pairs of estimates correspond to different age strata. Points indicate median, thick line segments indicate 50% posterior predictive intervals (PPIs), and thin lines indicate 95% PPIs. See Table S1 for more information about these studies. Results for other serology scenarios were similar and are not shown.

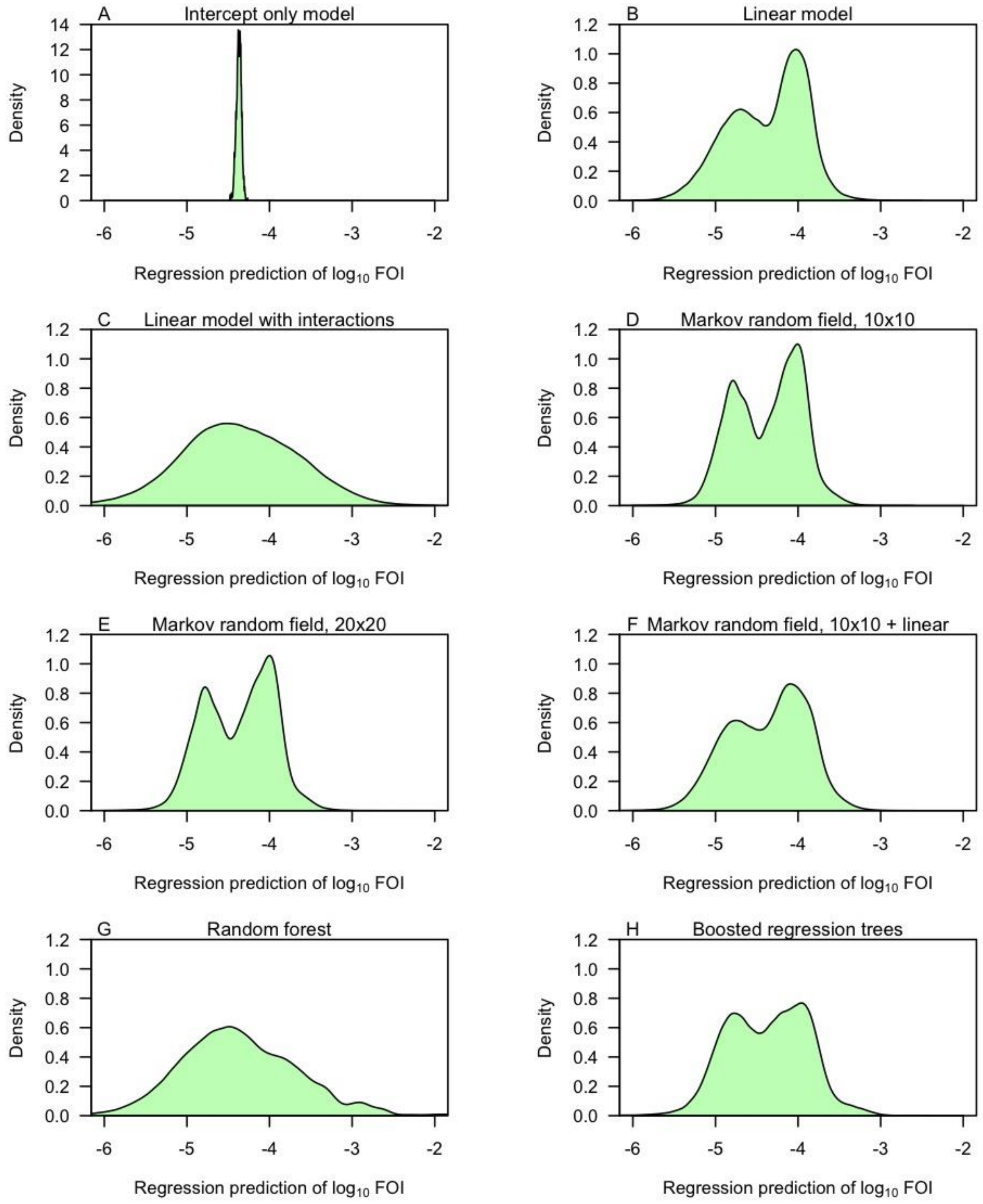

Figure S2. Distributions of predictions of force of infection from Step 4 (x-axis) under eight alternative regression models. These distributions pool predictions across all adm1s and reflect the full uncertainty in each model's predictions. Results shown here pertain to serology scenario 1 only.

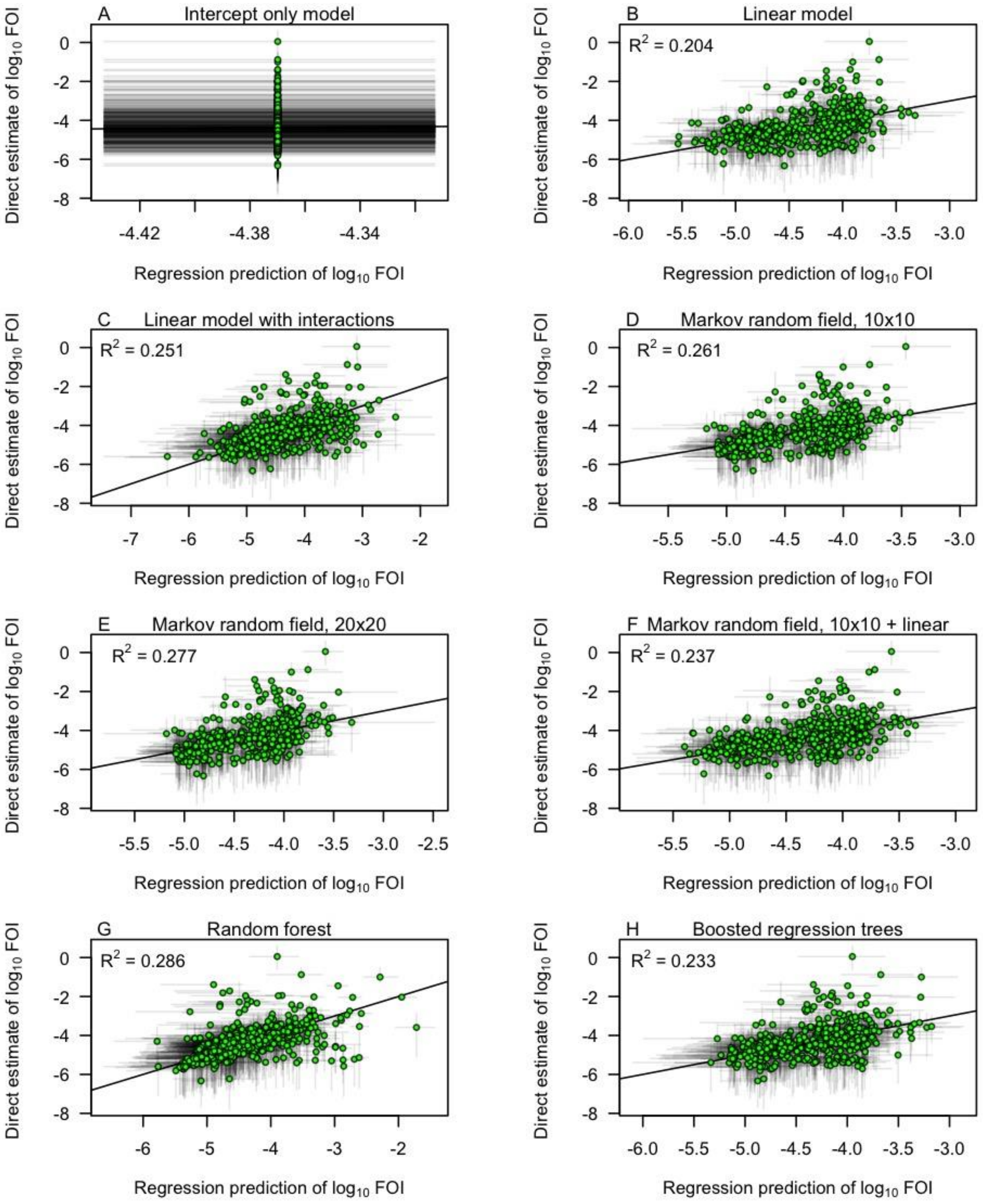

Figure S3. Comparison of regression predictions of force of infection from Step 4 (x-axis) against projected values from Step 3 (y-axis). Green circles indicate median values, and gray line segments indicate 95% uncertainty intervals. The coefficients of determination, R^2, in each panel were calculated based on median values.

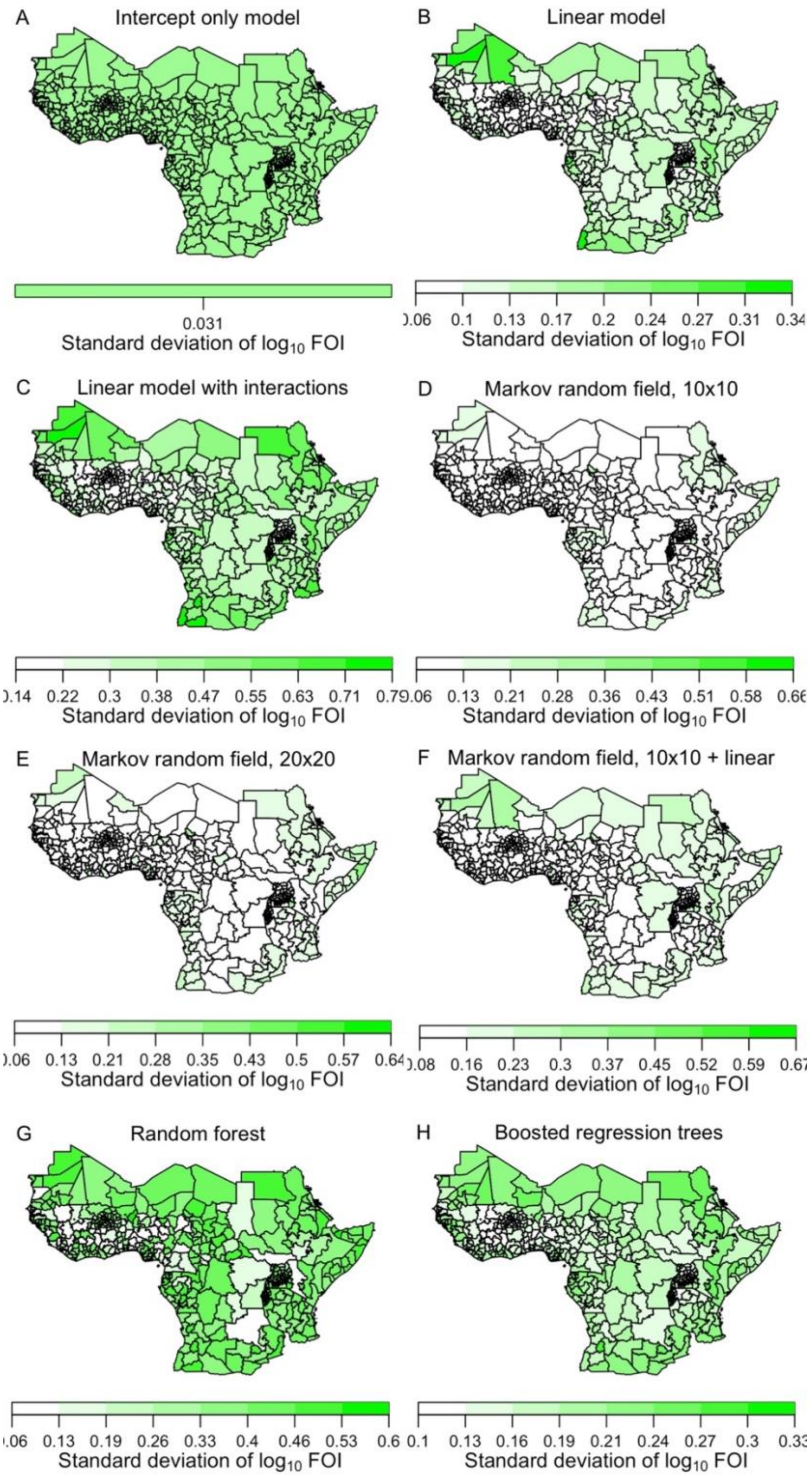

Figure S4. Uncertainty in spatial prediction of force of infection from eight regression models. Standard deviations of values on a $\log_{10}$ scale are shown from serology scenario 1. Color axes for each model differ so as to maximize contrast within each panel. Other serology scenarios produced similar results, but with magnitude varying according to differences in the estimated average reporting probabilities shown in Fig. 3.

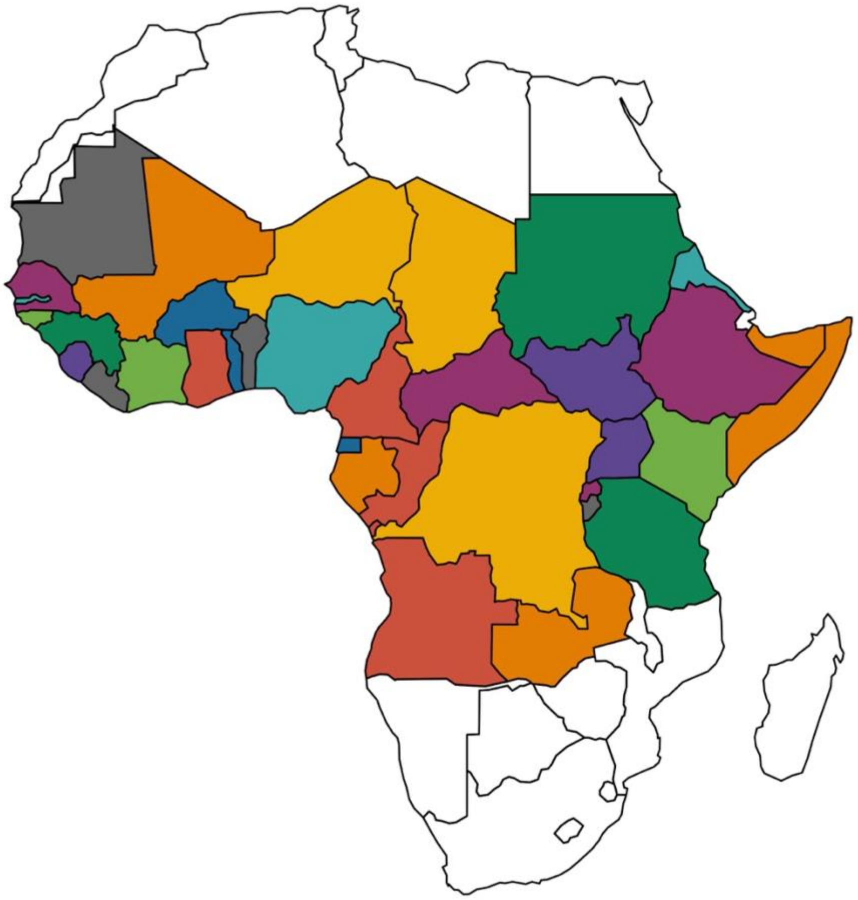

Figure S5. Country partitioning for cross-validation. Each color signifies a different set of countries for which data was withheld from model fitting and used to assess out-of-fit prediction. This partitioning was determined so as to maximize the evenness of the number of first-level administrative units (adm1s) across partitions. To do this, the first partition took the country with the most adm1s and the two countries with the fewest and grouped them together (i.e., Uganda, South Sudan, and Sierra Leone in purple). The second partition took the country with the second most adm1s and the two countries with the third and fourth fewest adm1s (i.e., Burkina Faso, Togo, and Equatorial Guinea in dark blue). This process was repeated until ten partitions of three to four countries each were obtained. Countries in white were not included in our analysis.

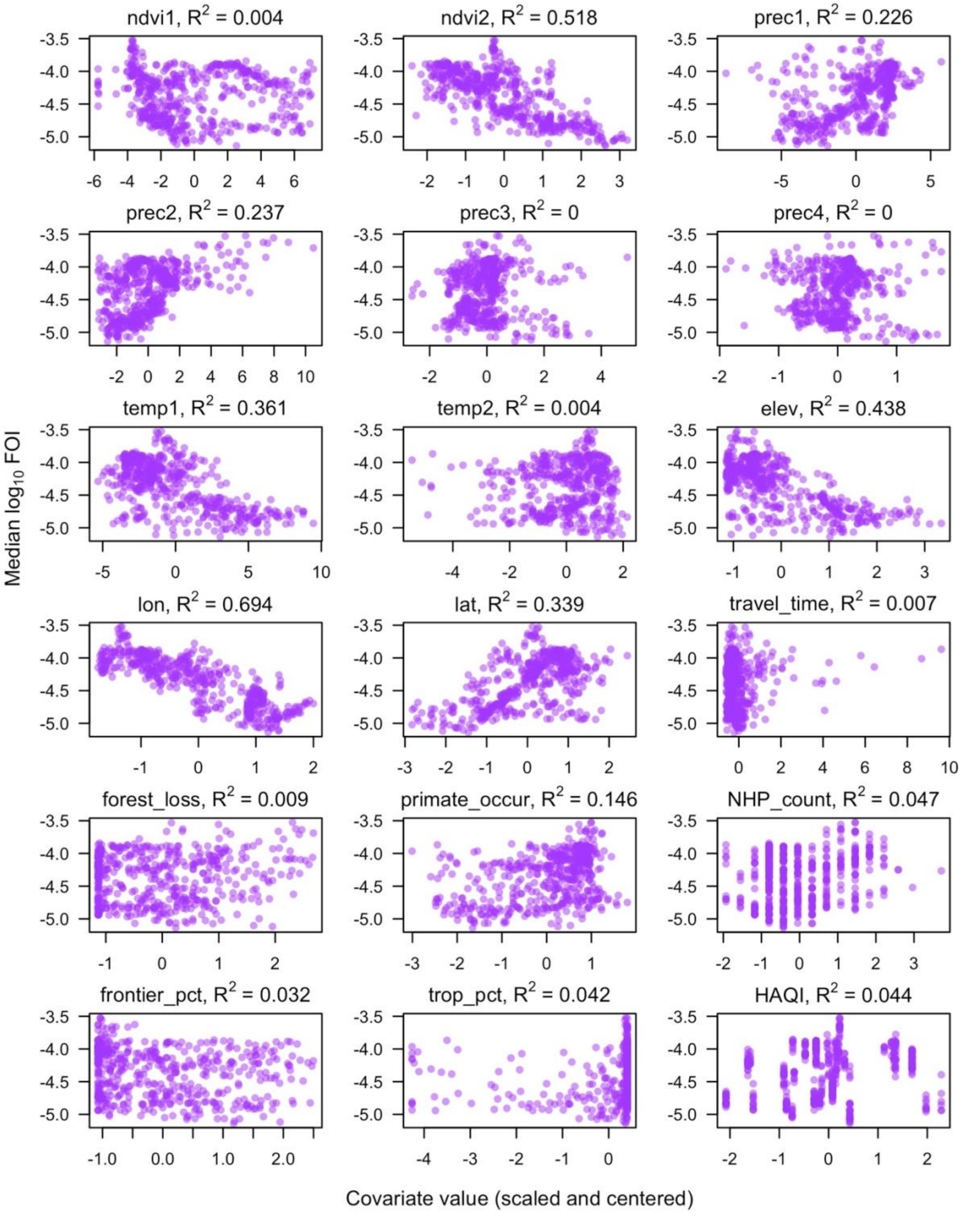

Figure S6. Associations between spatial covariates and $\log_{10}$ force of infection. Each dot corresponds to an adm1, with median values shown here and used to compute coefficients of determination, R^2. All spatial covariates were centered and scaled for this and other analyses.

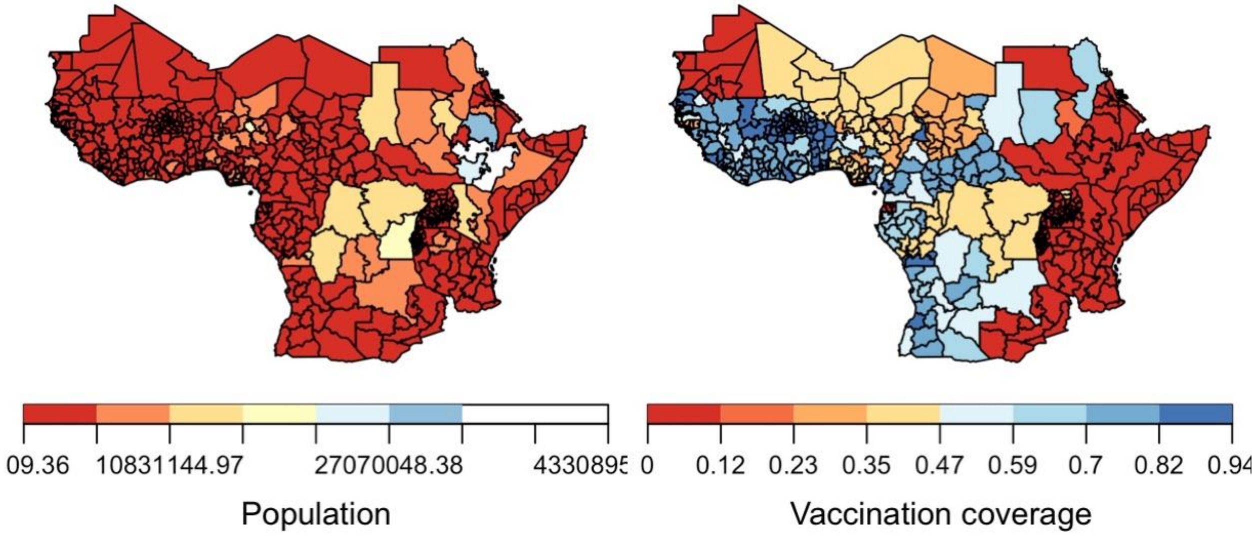

Figure S7. Population and vaccination coverage maps. Both maps reflect an average across 2021-2030. In the case of vaccination coverage, age-specific values of vaccination coverage were averaged proportion to population by age. All population and vaccination coverage estimates used in this analysis were generated by Hamlet et al. (*8*).

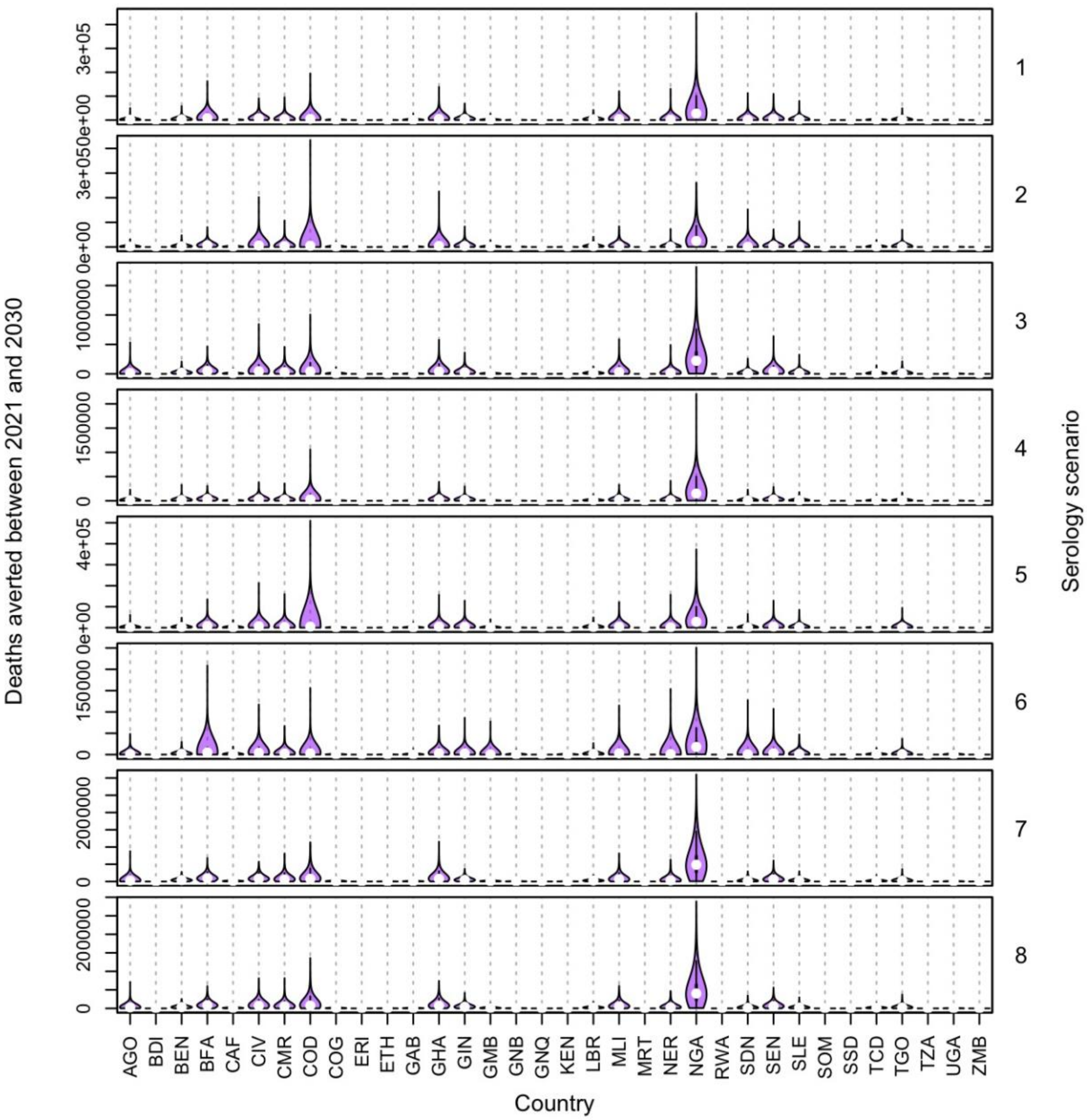

Figure S8. Deaths averted by country during 2021-2030. Results from the ensemble model corresponding to each serology scenario are shown in each panel.

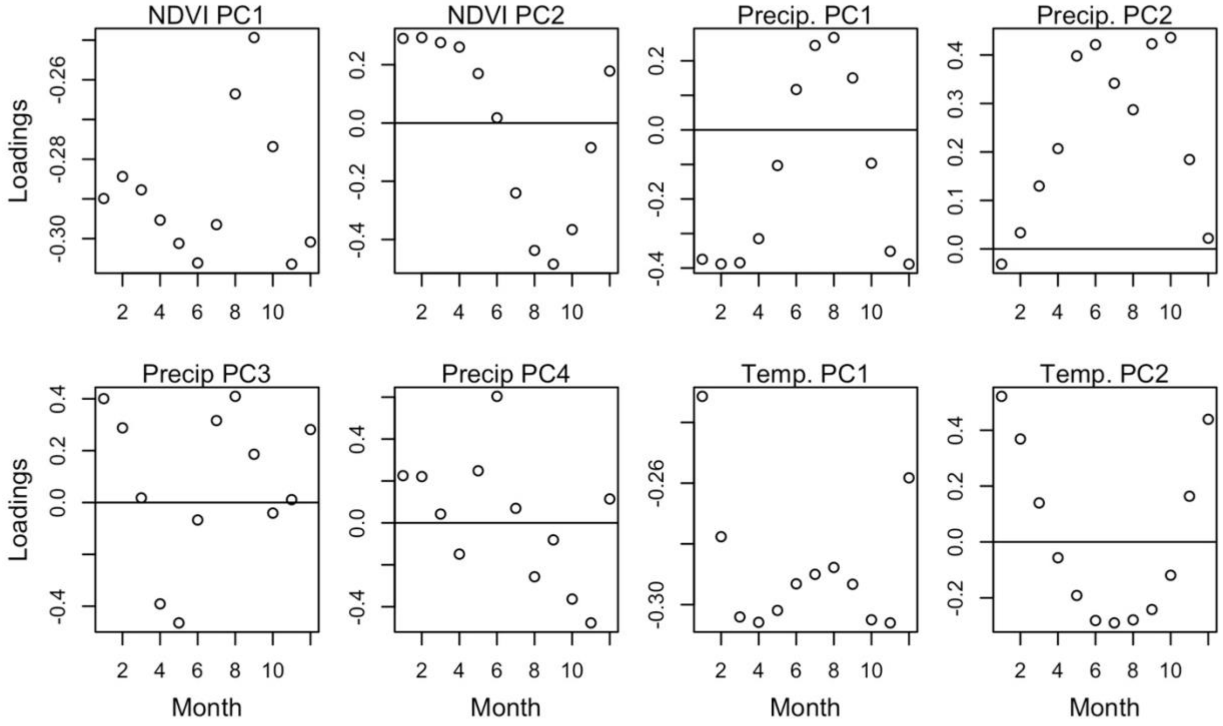

Figure S9. Principal component loadings for spatial covariates with monthly values. Each principal component results from summing the product of these loadings and the monthly values of a given spatial covariate for each administrative unit. For example, NDVI PC1 results from taking the loadings in the upper left panel, multiplying them by monthly NDVI values for each administrative unit, and then taking the corresponding sums. This reduces the dimensionality of the twelve monthly values of these three variables (a total of 36 variables) down to the eight represented here. The number of principal components for each of NDVI, precipitation, and temperature was chosen such that 95% of variation in those variables was accounted for by these principal components. Maps of the resulting principal components are displayed in Fig. S10. As can be gleaned from the loadings, many of these principal components appear to capture differences in seasonal climatic patterns across the study region.

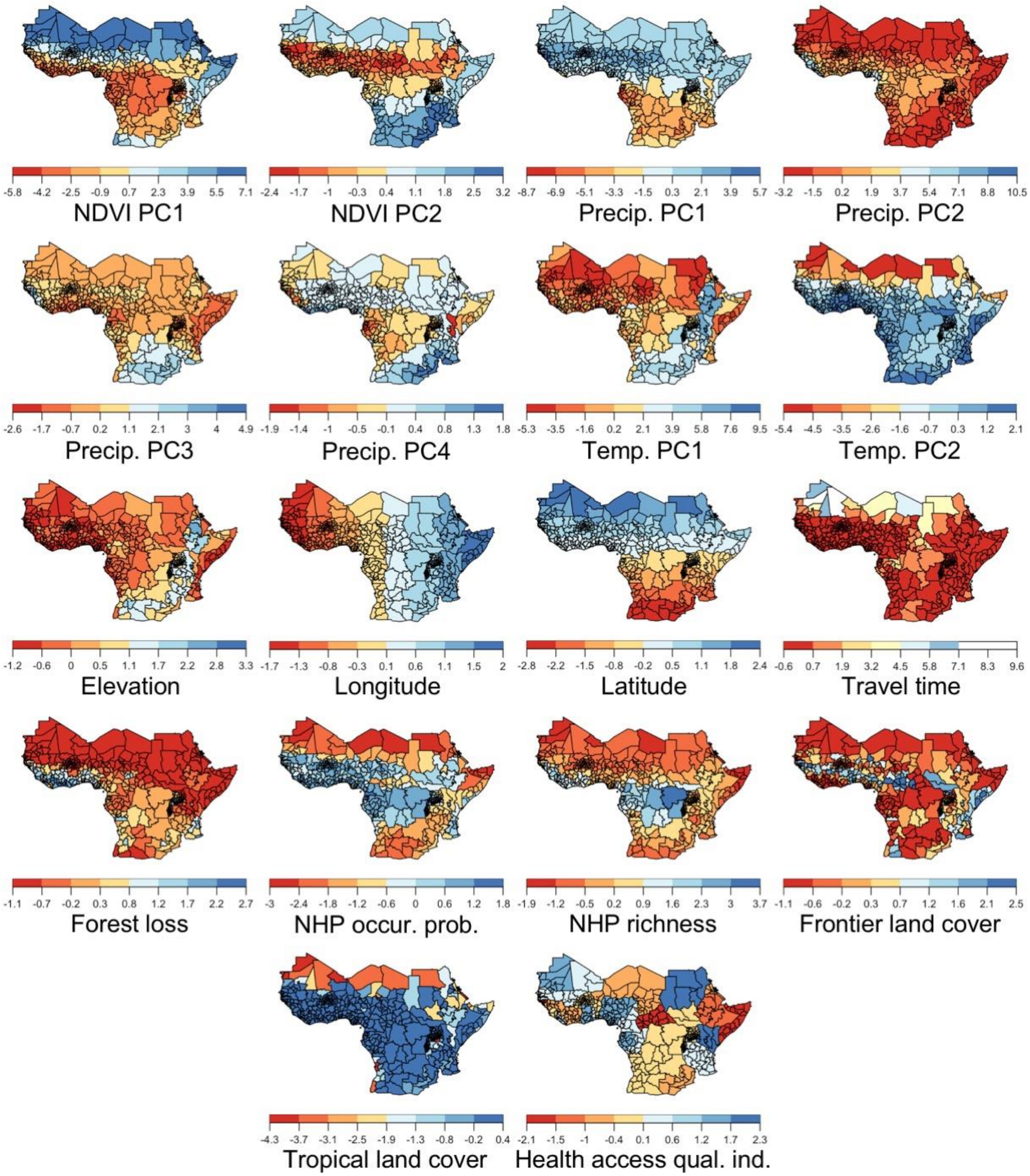

Figure S10. Spatial covariates used in the regression analysis in Step 4. All variables shown here have been centered and scaled, which is how they were used in the regression analysis and is sufficient to convey their relative spatial patterns. Rather than making inferences about relationships between these variables and force of infection, our goal was ensuring that we had variables with sufficiently diverse spatial patterns that the regression models would have sufficient flexibility to capture patterns in force of infection projected from Step 3.

Table S1. Characteristics of serological studies. Columns include characteristics that: allow for cross-referencing with Fig. 2, Fig. S1, and Fig. 3 (country, year, order therein); are related to how the serology scenarios were defined (vaccination status, vaccination coverage, outbreak investigation); determine force of infection (seropositive, number tested); and determine estimates of site-specific reporting probabilities (reported cases, deaths). Note that the number seropositive and number tested are totals across all age groups, which varied across studies and are not shown in this table in the interest of space. Vaccination coverage pertains to the year of the study and reflects a population-weighted average across age groups in the study.

Country	Year	Vacc. status	Vacc. cov.	Outbreak investig.	Sero. pos.	Sero. tested	Cases	Deaths	Ref.
CAF	2008	No	0.37	No	0	18	0	0	(*54*)
CAF	2008	No	0.39	No	48	339	0	0	(*54*)
CAF	2008	No	0.40	No	10	80	0	0	(*54*)
CAF	2008	No	0.39	No	18	112	0	0	(*54*)
CAF	2008	No	0.39	No	8	211	0	0	(*54*)
CAF	2008	No	0.44	No	11	64	2	0	(*54*)
CAF	2008	No	0.37	No	30	164	0	0	(*54*)
CMR	1984	No	0.20	Yes	10	90	3	2	(*55*)
CMR	1984	No	0.36	Yes	17	24	8	2	(*55*)
CMR	2000	No	0.28	No	13	55	4	0	(*56*)
CMR	2000	No	0.19	No	30	59	3	0	(*56*)
CMR	2000	No	0.19	No	13	85	0	0	(*56*)
CMR	2000	No	0.27	No	9	27	4	1	(*56*)
CMR	2000	No	0.19	No	4	30	2	0	(*56*)
ETH	2014	No	0.00	No	0	64	6	0	(*57*)
ETH	2014	No	0.00	No	0	135	0	0	(*57*)
ETH	2014	No	0.00	No	8	1,313	0	0	(*57*)
ETH	2014	No	0.00	No	2	152	0	0	(*57*)
KEN	2010	Unknown	0.06	No	22	433	119	46	(*58*)
KEN	2010	Unknown	0.01	No	6	36	0	0	(*58*)
NGA	1986	No	0.36	Yes	37	207	1,295	564	(*59*)
NGA	2008	No	0.29	No	25	310	407	46	(*60*)
SDN	2005	No	0.06	Yes	3	3	0	0	(*61*)

REFERENCES AND NOTES

1. T. P. Monath, P. F. C. Vasconcelos, Yellow fever. *J. Clin. Virol.* **64**, 160–173 (2015).
2. A. D. T. Barrett, S. Higgs, Yellow fever: A disease that has yet to be conquered. *Annu. Rev. Entomol.* **52**, 209–229 (2007).
3. M. A. Johansson, P. F. C. Vasconcelos, J. E. Staples, The whole iceberg: Estimating the incidence of yellow fever virus infection from the number of severe cases. *Trans. R. Soc. Trop. Med. Hyg.* **108**, 482–487 (2014).
4. K. Jean, C. A. Donnelly, N. M. Ferguson, T. Garske, A meta-analysis of serological response associated with yellow fever vaccination. *Am. J. Trop. Med. Hyg.* **95**, 1435–1439 (2016).
5. Eliminate yellow fever epidemics (EYE) 2017–2026; https://www.who.int/publications/i/item/9789241513661.
6. A. D. T. Barrett, Yellow fever in Angola and beyond—The problem of vaccine supply and demand. *N. Engl. J. Med.* **375**, 301–303 (2016).
7. F. M. Shearer, C. L. Moyes, D. M. Pigott, O. J. Brady, F. Marinho, A. Deshpande, J. Longbottom, A. J. Browne, M. U. G. Kraemer, K. M. O'Reilly, J. Hombach, S. Yactayo, V. E. M. de Araújo, A. A. da Nóbrega, J. F. Mosser, J. D. Stanaway, S. S. Lim, S. I. Hay, N. Golding, R. C. Reiner Jr, Global yellow fever vaccination coverage from 1970 to 2016: An adjusted retrospective analysis. *Lancet Infect. Dis.* **17**, 1209–1217 (2017).
8. A. Hamlet, K. Jean, S. Yactayo, J. Benzler, L. Cibrelus, N. Ferguson, T. Garske, POLICI: A web application for visualising and extracting yellow fever vaccination coverage in Africa. *Vaccine* **37**, 1384–1388 (2019).
9. T. Garske, M. D. Van Kerkhove, S. Yactayo, O. Ronveaux, R. F. Lewis, J. E. Staples, W. Perea, N. M. Ferguson; Yellow Fever Expert Committee, Yellow Fever in Africa: Estimating the burden of disease and impact of mass vaccination from outbreak and serological data. *PLOS Med.* **11**, e1001638 (2014).
10. K. Jean, A. Hamlet, J. Benzler, L. Cibrelus, K. A. M. Gaythorpe, A. Sall, N. M. Ferguson, T. Garske, Eliminating yellow fever epidemics in Africa: Vaccine demand forecast and impact modelling. *PLOS Negl. Trop. Dis.* **14**, e0008304 (2020).
11. K. A. Gaythorpe, A. Hamlet, L. Cibrelus, T. Garske, N. M. Ferguson, The effect of climate change on yellow fever disease burden in Africa. *eLife* **9**, e55619 (2020).

12. K. A. M. Gaythorpe, A. T. P. Hamlet, K. Jean, D. G. Ramos, L. Cibrelus, T. Garske, N. M. Ferguson, The global burden of yellow fever. *eLife* **10**, e64670 (2021).
13. P. N. Hamrick, S. Aldighieri, G. Machado, D. G. Leonel, L. M. Vilca, S. Uriona, M. C. Schneider, Geographic patterns and environmental factors associated with human yellow fever presence in the Americas. *PLOS Negl. Trop. Dis.* **11**, e0005897 (2017).
14. M. L. Childs, N. Nova, J. Colvin, E. A. Mordecai, Mosquito and primate ecology predict human risk of yellow fever virus spillover in Brazil. *Philos. Trans. R. Soc. Lond. B Biol. Sci.* **374**, 20180335 (2019).
15. M. A. B. de Almeida, E. Dos Santos, J. da C. Cardoso, L. G. da Silva, R. M. Rabelo, J. C. Bicca-Marques, Predicting Yellow fever through species distribution modeling of virus, vector, and monkeys. *Ecohealth* **16**, 95–108 (2019).
16. B. de Thoisy, N. I. O. Silva, L. Sacchetto, G. de Souza Trindade, B. P. Drumond, Spatial epidemiology of yellow fever: Identification of determinants of the 2016-2018 epidemics and at-risk areas in Brazil. *PLOS Negl. Trop. Dis.* **14**, e0008691 (2020).
17. F. M. Shearer, J. Longbottom, A. J. Browne, D. M. Pigott, O. J. Brady, M. U. G. Kraemer, F. Marinho, S. Yactayo, V. E. M. de Araújo, A. A. da Nóbrega, N. Fullman, S. E. Ray, J. F. Mosser, J. D. Stanaway, S. S. Lim, R. C. Reiner Jr, C. L. Moyes, S. I. Hay, N. Golding, Existing and potential infection risk zones of yellow fever worldwide: A modelling analysis. *Lancet Glob. Health* **6**, e270–e278 (2018).
18. K. A. M. Gaythorpe, K. Jean, L. Cibrelus, T. Garske, Quantifying model evidence for yellow fever transmission routes in Africa. *PLOS Comput. Biol.* **15**, e1007355 (2019).
19. A. Rachas, E. Nakouné, J. Bouscaillou, J. Paireau, B. Selekon, D. Senekian, A. Fontanet, M. Kazanji, Timeliness of yellow fever surveillance, Central African Republic. *Emerg. Infect. Dis.* **20**, 1004–1008 (2014).
20. C. L. Gardner, K. D. Ryman, Yellow fever: A reemerging threat. *Clin. Lab. Med.* **30**, 237–260 (2010).
21. J. J. Valadez, L. H. Weld, Maternal recall error of child vaccination status in a developing nation. *Am. J. Public Health* **82**, 120–122 (1992).
22. P. Bolton, E. Holt, A. Ross, N. Hughart, B. Guyer, Estimating vaccination coverage using parental recall, vaccination cards, and medical records. *Public Health Rep.* **113**, 521–526 (1998).

23. M. Miles, T. K. Ryman, V. Dietz, E. Zell, E. T. Luman, Validity of vaccination cards and parental recall to estimate vaccination coverage: A systematic review of the literature. *Vaccine* **31**, 1560–1568 (2013).
24. C. Domingo, R. N. Charrel, J. Schmidt-Chanasit, H. Zeller, C. Reusken, Yellow fever in the diagnostics laboratory. *Emerg. Microbes Infect.* **7**, 129 (2018).
25. X. Li, C. Mukandavire, Z. M. Cucunubá, K. Abbas, H. E. Clapham, M. Jit, H. L. Johnson, T. Papadopoulos, E. Vynnycky, M. Brisson, E. D. Carter, A. D. Clark, M. J. de Villiers, K. Eilertson, M. J. Ferrari, I. Gamkrelidze, K. Gaythorpe, N. C. Grassly, T. B. Hallett, W. Hinsley, M. L. Jackson, K. Jean, A. Karachaliou, P. Klepac, J. Lessler, X. Li, S. M. Moore, S. Nayagam, D. M. Nguyen, H. Razavi, D. Razavi-Shearer, S. Resch, C. Sanderson, S. Sweet, S. Sy, Y. Tam, H. Tanvir, Q. M. Tran, C. L. Trotter, S. Truelove, K. Zandvoort, S. Verguet, N. Walker, A. Winter, K. Woodruff, N. M. Ferguson, T. Garske; Vaccine Impact Modelling Consortium, Estimating the health impact of vaccination against 10 pathogens in 98 low and middle income countries from 2000 to 2030. *medRxiv* 10.2139/ssrn.3460657 (2019).
26. S. Bhatt, E. Cameron, S. R. Flaxman, D. J. Weiss, D. L. Smith, P. W. Gething, Improved prediction accuracy for disease risk mapping using Gaussian process stacked generalization. *J. R. Soc. Interface* **14**, 20170520 (2017).
27. R. C. Reiner, C. A. Welgan, D. C. Casey, C. E. Troeger, M. M. Baumann, Q.A. P. Nguyen, S. J. Swartz, B. F. Blacker, A. Deshpande, J. F. Mosser, A. E. Osgood-Zimmerman, L. Earl, L. B. Marczak, S. B. Munro, M. K. Miller-Petrie, G. Rodgers Kemp, J. Frostad, K. E. Wiens, P. A. Lindstedt, D. M. Pigott, L. Dwyer-Lindgren, J. M. Ross, R. Burstein, N. Graetz, P. C. Rao, I. A. Khalil, N. Davis Weaver, S. E. Ray, I. Davis, T. Farag, O. J. Brady, M. U. G. Kraemer, D. L. Smith, S. Bhatt, D. J. Weiss, P. W. Gething, N. J. Kassebaum, A. H. Mokdad, C. J. L. Murray, S. I. Hay, Identifying residual hotspots and mapping lower respiratory infection morbidity and mortality in African children from 2000 to 2017. *Nat. Microbiol.* **4**, 2310–2318 (2019).
28. L. Dwyer-Lindgren, M. A. Cork, A. Sligar, K. M. Steuben, K. F. Wilson, N. R. Provost, B. K. Mayala, J. D. VanderHeide, M. L. Collison, J. B. Hall, M. H. Biehl, A. Carter, T. Frank, D. Douwes-Schultz, R. Burstein, D. C. Casey, A. Deshpande, L. Earl, C. el Bcheraoui, T. H. Farag, N. J. Henry, D. Kinyoki, L. B. Marczak, M. R. Nixon, A. Osgood-Zimmerman, D. Pigott, R. C. Reiner Jr, J. M. Ross, L. E. Schaeffer, D. L. Smith, N. Davis Weaver, K. E. Wiens, J. W. Eaton, J. E. Justman, A.

Opio, B. Sartorius, F. Tanser, N. Wabiri, P. Piot, C. J. L. Murray, S. I. Hay, Mapping HIV prevalence in sub-Saharan Africa between 2000 and 2017. *Nature* **570**, 189–193 (2019).

29. T. P. Van Boeckel, J. Pires, R. Silvester, C. Zhao, J. Song, N. G. Criscuolo, M. Gilbert, S. Bonhoeffer, R. Laxminarayan, Global trends in antimicrobial resistance in animals in low- and middle-income countries. *Science* **365**, 6459 (2019).
30. Local Burden of Disease Diarrhoea Collaborators, Mapping geographical inequalities in childhood diarrhoeal morbidity and mortality in low-income and middle-income countries, 2000–17: Analysis for the Global Burden of Disease Study 2017. *Lancet* **395**, 1779–1801 (2020).
31. G. Chowell, R. Luo, K. Sun, K. Roosa, A. Tariq, C. Viboud, Real-time forecasting of epidemic trajectories using computational dynamic ensembles. *Epidemics* **30**, 100379 (2020).
32. K. Shea, M. C. Runge, D. Pannell, W. J. M. Probert, S.-L. Li, M. Tildesley, M. Ferrari, Harnessing multiple models for outbreak management. *Science* **368**, 577–579 (2020).
33. N. E. Dean, A. P. Y. Piontti, Z. J. Madewell, D. A. T. Cummings, M. D. T. Hitchings, K. Joshi, R. Kahn, A. Vespignani, M. E. Halloran, I. M. Longini Jr, Ensemble forecast modeling for the design of COVID-19 vaccine efficacy trials. *Vaccine* **38**, 7213–7216 (2020).
34. E. L. Ray, N. Wattanachit, J. Niemi, A. H. Kanji, K. House, E. Y. Cramer, J. Bracher, A. Zheng, T. K. Yamana, X. Xiong, S. Woody, Y. Wang, L. Wang, R. L. Walraven, V. Tomar, K. Sherratt, D. Sheldon, R. C. Reiner Jr, B. Aditya Prakash, D. Osthus, M. L. Li, E. C. Lee, U. Koyluoglu, P. Keskinocak, Y. Gu, Q. Gu, G. E. George, G. España, S. Corsetti, View ORCID Profile Jagpreet Chhatwal, S. Cavany, H. Biegel, M. Ben-Nun, J. Walker, R. Slayton, V. Lopez, M. Biggerstaff, M. A. Johansson, N. G. Reich, Ensemble forecasts of coronavirus disease 2019 (COVID-19) in the U.S. *medRxiv* 10.1101/2020.08.19.20177493 (2020).
35. World Health Organization, *Eliminate Yellow Fever Epidemics (EYE): Strategy Partners Meeting Report: Geneva, Switzerland, 9–10 May 2017* (World Health Organization, 2018); https://apps.who.int/iris/bitstream/handle/10665/279723/WHO-WHE-IHM-2018.5-eng.pdf.
36. M. U. G. Kraemer, N. R. Faria, R. C. Reiner Jr, N. Golding, B. Nikolay, S. Stasse, M. A. Johansson, H. Salje, O. Faye, G. R. W. Wint, M. Niedrig, F. M. Shearer, S. C. Hill, R. N. Thompson, D. Bisanzio, N. Taveira, H. H. Nax, B. S. R. Pradelski, E. O. Nsoesie, N. R. Murphy, I. I. Bogoch, K. Khan, J. S. Brownstein, A. J. Tatem, T. de Oliveira, D. L. Smith, A. A. Sall, O. G. Pybus, S. I. Hay, S. Cauchemez, Spread of yellow fever virus outbreak in Angola and the Democratic Republic of the Congo 2015-16: A modelling study. *Lancet Infect. Dis.* **17**, 330–338 (2017).

37. S. Zhao, L. Stone, D. Gao, D. He, Modelling the large-scale yellow fever outbreak in Luanda, Angola, and the impact of vaccination. *PLOS Negl. Trop. Dis.* **12**, e0006158 (2018).
38. A. F. Brito, L. C. Machado, M. J. L. Siconelli, R. J. Oidtman, J. R. Fauver, R. D. de Oliveira Carvalho, F. Z. Dezordi, M. R. Pereira, L. A. de Castro-Jorge, E. C. M. Minto, L. M. R. Passos, C. C. Kalinich, M. E. Petrone, E. Allen, G. C. España, A. T. Huang, D. A. T. Cummings, G. Baele, R. F. O. Franca, T. Alex Perkins, B. A. Lopes da Fonseca, G. L. Wallau, N. D. Grubaugh, Lying in wait: The resurgence of dengue virus after the Zika epidemic in Brazil. *Nat. Commun.* **12**, 2619 (2021).
39. S. E. Robertson, B. P. Hull, O. Tomori, O. Bele, J. W. LeDuc, K. Esteves, Yellow fever: A decade of reemergence. *JAMA* **276**, 1157–1162 (1996).
40. Collaborative group for studies on yellow fever vaccines, Duration of immunity in recipients of two doses of 17DD yellow fever vaccine. *Vaccine* **37**, 5129–5135 (2019).
41. C. Domingo, J. Fraissinet, P. O. Ansah, C. Kelly, N. Bhat, S. O. Sow, J. E. Mejía, Long-term immunity against yellow fever in children vaccinated during infancy: A longitudinal cohort study. *Lancet Infect. Dis.* **19**, 1363–1370 (2019).
42. J. E. Staples, A. D. T. Barrett, A. Wilder-Smith, J. Hombach, Review of data and knowledge gaps regarding yellow fever vaccine-induced immunity and duration of protection. *NPJ Vaccines* **5**, 54 (2020).
43. United Nations Department of Economic and Social Affairs, *World Population Prospects* (United Nations Department of Economic and Social Affairs, 2016); 10.18356/a3bacd57-en.
44. E. A. Bright, P. R. Coleman, A. N. Rose, M. L. Urban, *LandScan* (Oak Ridge National Laboratory, 2014).
45. LP DAAC—MOD13A3 (2018); https://lpdaac.usgs.gov/products/mod13a3v006/.
46. R. J. Hijmans, S. E. Cameron, J. L. Parra, P. G. Jones, A. Jarvis, Very high resolution interpolated climate surfaces for global land areas. *Int. J. Climatol.* **25**, 1965–1978 (2005).
47. Earth Resources Observation and Science Center/U.S. Geological Survey/U.S. Department of the Interior, *USGS 30 ARC-second Global Elevation Data, GTOPO30* (Research Data Archive at the National Center for Atmospheric Research, Computational and Information Systems Laboratory, 1997).
48. D. J. Weiss, A. Nelson, H. S. Gibson, W. Temperley, S. Peedell, A. Lieber, M. Hancher, E. Poyart, S. Belchior, N. Fullman, B. Mappin, U. Dalrymple, J. Rozier, T. C. D. Lucas, R. E. Howes, L. S.

Tusting, S. Y. Kang, E. Cameron, D. Bisanzio, K. E. Battle, S. Bhatt, P. W. Gething, A global map of travel time to cities to assess inequalities in accessibility in 2015. *Nature* **553**, 333–336 (2018).

49. *2014 IUCN Annual Report* (IUCN, 2015).
50. D. M. Olson, E. Dinerstein, E. D. Wikramanayake, N. D. Burgess, G. V. N. Powell, E. C. Underwood, J. A. D'amico, I. Itoua, H. E. Strand, J. C. Morrison, C. J. Loucks, T. F. Allnutt, T. H. Ricketts, Y. Kura, J. F. Lamoreux, W. W. Wettengel, P. Hedao, K. R. Kassem, Terrestrial ecoregions of the world: A new map of life on Earth. *Bioscience* **51**, 933 (2001).
51. M. C. Hansen, P. V. Potapov, R. Moore, M. Hancher, S. A. Turubanova, A. Tyukavina, D. Thau, S. V. Stehman, S. J. Goetz, T. R. Loveland, A. Kommareddy, A. Egorov, L. Chini, C. O. Justice, J. R. G. Townshend, High-resolution global maps of 21st-century forest cover change. *Science* **342**, 850–853 (2013).
52. N. Fullman, J. Yearwood, S. M. Abay, C. Abbafati, F. Abd-Allah, J. Abdela, A. Abdelalim, Z. Abebe, T. A. Abebo, V. Aboyans, H. N. Abraha, D. M. X. Abreu, L. J. Abu-Raddad, A. A. Adane, R. A. Adedoyin, O. Adetokunboh, T. B. Adhikari, M. Afarideh, A. Afshin, G. Agarwal, D. Agius, A. Agrawal, S. Agrawal, A. Ahmad Kiadaliri, M. T. E. Aichour, M. Akibu, R. O. Akinyemi, T. F. Akinyemiju, N. Akseer, F. H. al Lami, F. Alahdab, Z. al-Aly, K. Alam, T. Alam, D. Alasfoor, M. I. Albittar, K. A. Alene, A. al-Eyadhy, S. D. Ali, M. Alijanzadeh, S. M. Aljunid, A.'. Alkerwi, F. Alla, P. Allebeck, C. Allen, M. A. Alomari, R. al-Raddadi, U. Alsharif, K. A. Altirkawi, N. Alvis-Guzman, A. T. Amare, K. Amenu, W. Ammar, Y. A. Amoako, N. Anber, C. L. Andrei, S. Androudi, C. A. T. Antonio, V. E. M. Araújo, O. Aremu, J. Ärnlöv, A. Artaman, K. K. Aryal, H. Asayesh, E. T. Asfaw, S. W. Asgedom, R. J. Asghar, M. M. Ashebir, N. A. Asseffa, T. M. Atey, S. R. Atre, M. S. Atteraya, L. Avila-Burgos, E. F. G. A. Avokpaho, A. Awasthi, B. P. Ayala Quintanilla, A. A. Ayalew, H. T. Ayele, R. Ayer, T. B. Ayuk, P. Azzopardi, N. Azzopardi-Muscat, T. K. Babalola, H. Badali, A. Badawi, M. Banach, A. Banerjee, A. Banstola, R. M. Barber, M. A. Barboza, S. L. Barker-Collo, T. Bärnighausen, S. Barquera, L. H. Barrero, Q. Bassat, S. Basu, B. T. Baune, S. Bazargan-Hejazi, N. Bedi, E. Beghi, M. Behzadifar, M. Behzadifar, B. B. Bekele, A. B. Belachew, S. A. Belay, Y. A. Belay, M. L. Bell, A. K. Bello, D. A. Bennett, J. R. Bennett, I. M. Bensenor, D. F. Berhe, E. Bernabé, R. S. Bernstein, M. Beuran, A. Bhalla, P. Bhatt, S. Bhaumik, Z. A. Bhutta, B. Biadgo, A. Bijani, B. Bikbov, C. Birungi, S. Biryukov, H. Bizuneh, I. W. Bolliger, K. Bolt, I. R. Bou-Orm, K. Bozorgmehr, O. J. Brady, A. Brazinova, N. J. K. Breitborde, H. Brenner, G. Britton, T. S. Brugha, Z. A. Butt, L. Cahuana-Hurtado, I. R. Campos-Nonato, J. C. Campuzano, J. Car, M. Car,

R. Cárdenas, J. J. Carrero, F. Carvalho, C. A. Castañeda-Orjuela, J. Castillo Rivas, F. Catalá-López, K. Cercy, J. Chalek, H.Y. Chang, J.C. Chang, A. Chattopadhyay, P. Chaturvedi, P. P.C. Chiang, V. H. Chisumpa, J.Y. J. Choi, H. Christensen, D. J. Christopher, S.C. Chung, L. G. Ciobanu, M. Cirillo, D. Colombara, S. Conti, C. Cooper, L. Cornaby, P. A. Cortesi, M. Cortinovis, A. Costa Pereira, E. Cousin, M. H. Criqui, E. A. Cromwell, C. S. Crowe, J. A. Crump, A. K. Daba, B. A. Dachew, A. F. Dadi, L. Dandona, R. Dandona, P. I. Dargan, A. Daryani, M. Daryani, J. Das, S. K. Das, J. das Neves, N. Davis Weaver, K. Davletov, B. de Courten, D. de Leo, J.W. de Neve, R. P. Dellavalle, G. Demoz, K. Deribe, D. C. Des Jarlais, S. Dey, S. D. Dharmaratne, M. Dhimal, S. Djalalinia, D. T. Doku, K. Dolan, E. R. Dorsey, K. P. B. dos Santos, K. E. Doyle, T. R. Driscoll, M. Dubey, E. Dubljanin, B. B. Duncan, M. Echko, D. Edessa, D. Edvardsson, J. R. Ehrlich, E. Eldrenkamp, Z. Z. el-Khatib, M. Endres, A. Y. Endries, B. Eshrati, S. Eskandarieh, A. Esteghamati, M. Fakhar, T. Farag, M. Faramarzi, E. J. A. Faraon, A. Faro, F. Farzadfar, A. Fatusi, M. S. Fazeli, V. L. Feigin, A. B. Feigl, N. Fentahun, S.M. Fereshtehnejad, E. Fernandes, J. C. Fernandes, D. O. Fijabi, I. Filip, F. Fischer, C. Fitzmaurice, A. D. Flaxman, L. S. Flor, N. Foigt, K. J. Foreman, J. J. Frostad, T. Fürst, N. D. Futran, E. Gakidou, S. Gallus, K. Gambashidze, A. Gamkrelidze, M. Ganji, A. K. Gebre, T. T. Gebrehiwot, A. T. Gebremedhin, Y. A. Gelaw, J. M. Geleijnse, D. Geremew, P. W. Gething, R. Ghadimi, K. Ghasemi Falavarjani, M. Ghasemi-Kasman, P. S. Gill, A. Z. Giref, M. Giroud, M. D. Gishu, G. Giussani, W. W. Godwin, S. Goli, H. Gomez-Dantes, P. N. Gona, A. Goodridge, S. V. Gopalani, Y. Goryakin, A. C. Goulart, A. Grada, M. Griswold, G. Grosso, H. C. Gugnani, Y. Guo, R. Gupta, R. Gupta, T. Gupta, T. Gupta, V. Gupta, J. A. Haagsma, V. Hachinski, N. Hafezi-Nejad, G. B. Hailu, R. R. Hamadeh, S. Hamidi, G. J. Hankey, H. L. Harb, H. C. Harewood, S. Harikrishnan, J. M. Haro, H. Y. Hassen, R. Havmoeller, C. Hawley, S. I. Hay, J. He, S. J. C. Hearps, M. I. Hegazy, B. Heibati, M. Heidari, D. Hendrie, N. J. Henry, V. H. Herrera Ballesteros, C. Herteliu, D. T. Hibstu, M. K. Hiluf, H. W. Hoek, E. Homaie Rad, N. Horita, H. D. Hosgood, M. Hosseini, S. R. Hosseini, M. Hostiuc, S. Hostiuc, D. G. Hoy, M. Hsairi, A. S. Htet, G. Hu, J. J. Huang, K. M. Iburg, F. Idris, E. U. Igumbor, C. Ikeda, B. V. Ileanu, O. S. Ilesanmi, K. Innos, S. S. N. Irvani, C. M. S. Irvine, F. Islami, T. A. Jacobs, K. H. Jacobsen, N. Jahanmehr, R. Jain, S. K. Jain, M. B. Jakovljevic, M. T. Jalu, A. A. Jamal, M. Javanbakht, A. U. Jayatilleke, P. Jeemon, R. P. Jha, V. Jha, J. Jóúwiak, O. John, S. C. Johnson, J. B. Jonas, V. Joshua, M. Jürisson, Z. Kabir, R. Kadel, A. Kahsay, R. Kalani, C. Kar, M. Karanikolos, A. Karch, C. K. Karema, S. M. Karimi, A. Kasaeian, D. H. Kassa, G. M. Kassa, T. D. Kassa, N. J. Kassebaum, S. V. Katikireddi, A. Kaul, N. Kawakami, K. Kazanjan, S.

Kebede, P. N. Keiyoro, G. R. Kemp, A. P. Kengne, M. Kereselidze, E. B. Ketema, Y. S. Khader, M. A. Khafaie, A. Khajavi, I. A. Khalil, E. A. Khan, G. Khan, M. N. Khan, M. A. Khan, M. N. Khanal, Y.H. Khang, M. M. Khater, A. T. A. Khoja, A. Khosravi, J. Khubchandani, G. D. Kibret, D. N. Kiirithio, D. Kim, Y. J. Kim, R. W. Kimokoti, Y. Kinfu, S. Kinra, A. Kisa, N. Kissoon, S. Kochhar, Y. Kokubo, J. A. Kopec, S. Kosen, P. A. Koul, A. Koyanagi, M. Kravchenko, K. Krishan, K. J. Krohn, B. Kuate Defo, G. A. Kumar, P. Kumar, M. Kutz, I. Kuzin, H. H. Kyu, D. P. Lad, A. Lafranconi, D. K. Lal, R. Lalloo, H. Lam, Q. Lan, J. J. Lang, V. C. Lansingh, S. Lansky, A. Larsson, A. Latifi, J. V. Lazarus, J. L. Leasher, P. H. Lee, Y. Legesse, J. Leigh, C. T. Leshargie, S. Leta, J. Leung, R. Leung, M. Levi, Y. Li, J. Liang, M. L. Liben, L.L. Lim, S. S. Lim, M. Lind, S. Linn, S. Listl, P. Liu, S. Liu, R. Lodha, A. D. Lopez, S. A. Lorch, S. Lorkowski, P. A. Lotufo, T. C. D. Lucas, R. Lunevicius, G. Lurton, R. A. Lyons, F. Maalouf, E. R. K. Macarayan, M. T. Mackay, E. R. Maddison, F. Madotto, H. Magdy Abd el Razek, M. Magdy Abd el Razek, M. Majdan, R. Majdzadeh, A. Majeed, R. Malekzadeh, R. Malhotra, D. C. Malta, A. A. Mamun, T. Manhertz, H. Manguerra, M. A. Mansournia, L. G. Mantovani, T. Manyazewal, C. C. Mapoma, C. Margono, J. Martinez-Raga, S. C. O. Martins, F. R. Martins-Melo, I. Martopullo, W. März, B. B. Massenburg, M. R. Mathur, P. K. Maulik, M. Mazidi, C. McAlinden, J. J. McGrath, M. McKee, S. Mehata, R. Mehrotra, K. M. Mehta, V. Mehta, T. Meier, F. Mejia-Rodriguez, K. G. Meles, M. Melku, P. Memiah, Z. A. Memish, W. Mendoza, D. A. Mengiste, D. T. Mengistu, B. G. Menota, G. A. Mensah, A. Meretoja, T. J. Meretoja, H. B. Mezgebe, T. Miazgowski, R. Micha, R. Milam, A. Millear, T. R. Miller, G.K. Mini, S. Minnig, A. Mirica, E. M. Mirrakhimov, A. Misganaw, P. B. Mitchell, F. W. Mlashu, B. Moazen, K. A. Mohammad, R. Mohammadibakhsh, E. Mohammed, M. A. Mohammed, S. Mohammed, A. H. Mokdad, G. L. Mola, M. Molokhia, F. Momeniha, L. Monasta, J. C. Montañez Hernandez, M. Moosazadeh, M. Moradi-Lakeh, P. Moraga, L. Morawska, I. Moreno Velasquez, R. Mori, S. D. Morrison, M. Moses, S. M. Mousavi, U. O. Mueller, M. Murhekar, G. V. S. Murthy, S. Murthy, J. Musa, K. I. Musa, G. Mustafa, S. Muthupandian, C. Nagata, G. Nagel, M. Naghavi, A. Naheed, G. A. Naik, N. Naik, F. Najafi, L. Naldi, V. Nangia, J. R. N. Nansseu, K.M. V. Narayan, B. R. Nascimento, I. Negoi, R. I. Negoi, C. R. Newton, J. W. Ngunjiri, G. Nguyen, L. Nguyen, T. H. Nguyen, E. Nichols, D. N. A. Ningrum, E. Nolte, V. M. Nong, O. F. Norheim, B. Norrving, J. J. N. Noubiap, A. Nyandwi, C. M. Obermeyer, R. Ofori-Asenso, F. A. Ogbo, I.H. Oh, O. Oladimeji, A. T. Olagunju, T. O. Olagunju, P. R. Olivares, P. P. V. Oliveira, H. E. Olsen, B. O. Olusanya, J. O. Olusanya, K. Ong, J. N. Opio, E. Oren, D. V. Ortega-

Altamirano, A. Ortiz, R. Ozdemir, M. PA, A. W. Pain, M. R. T. Palone, A. Pana, S. Panda-Jonas, J. D. Pandian, E.K. Park, H. Parsian, T. Patel, S. Pati, S. T. Patil, A. Patle, G. C. Patton, V. R. Paturi, D. Paudel, M. M. Pedroso, S. P. Pedroza, D. M. Pereira, N. Perico, H. Peterson, M. Petzold, N. Peykari, M. R. Phillips, F. B. Piel, D. M. Pigott, J. D. Pillay, M. A. Piradov, S. Polinder, C. D. Pond, M. J. Postma, F. Pourmalek, S. Prakash, V. Prakash, N. Prasad, N. M. Prasad, C. Purcell, M. Qorbani, H. K. Quintana, A. Radfar, A. Rafay, A. Rafiei, K. Rahimi, A. Rahimi-Movaghar, V. Rahimi-Movaghar, M. Rahman, M. A. Rahman, S. U. Rahman, R. K. Rai, S. B. Raju, U. Ram, S. M. Rana, Z. Rankin, D. Rasella, D. L. Rawaf, S. Rawaf, S. E. Ray, C. A. Razo-García, P. Reddy, R. C. Reiner, C. Reis, M. B. Reitsma, G. Remuzzi, A. M. N. Renzaho, S. Resnikoff, S. Rezaei, M. S. Rezai, A. L. Ribeiro, M. J. Rios Blancas, J. A. Rivera, L. Roever, L. Ronfani, G. Roshandel, A. Rostami, G. A. Roth, D. Rothenbacher, A. Roy, N. Roy, G. M. Ruhago, Y. D. Sabde, P. S. Sachdev, N. Sadat, M. Safdarian, S. Safiri, R. Sagar, A. Sahebkar, M. A. Sahraian, H. S. Sajadi, J. Salama, P. Salamati, R. F. Saldanha, H. Salimzadeh, J. A. Salomon, A. M. Samy, J. R. Sanabria, P. K. Sancheti, M. D. Sanchez-Niño, D. Santomauro, I. S. Santos, M. M. Santric Milicevic, A. R. Sarker, N. Sarrafzadegan, B. Sartorius, M. Satpathy, M. Savic, M. Sawhney, S. Saxena, M. I. Saylan, E. Schaeffner, J. Schmidhuber, M. I. Schmidt, I. J. C. Schneider, A. E. Schumacher, A. E. Schutte, D. C. Schwebel, F. Schwendicke, M. Sekerija, S. G. Sepanlou, E. E. Servan-Mori, A. Shafieesabet, M. A. Shaikh, M. Shakh-Nazarova, M. Shams-Beyranvand, H. Sharafi, M. Sharif-Alhoseini, S. M. Shariful Islam, M. Sharma, R. Sharma, J. She, A. Sheikh, M. T. Shfare, P. Shi, C. Shields, M. Shigematsu, Y. Shinohara, R. Shiri, R. Shirkoohi, I. Shiue, M. G. Shrime, S. R. Shukla, S. Siabani, I. D. Sigfusdottir, D. H. Silberberg, D. A. S. Silva, J. P. Silva, D. G. A. Silveira, J. A. Singh, L. Singh, N. P. Singh, V. Singh, D. N. Sinha, A. H. Sinke, M. Sisay, V. Skirbekk, K. Sliwa, A. Smith, A. M. Soares Filho, B. H. A. Sobaih, M. Somai, S. Soneji, M. Soofi, R. J. D. Sorensen, J. B. Soriano, I. N. Soyiri, L. A. Sposato, C. T. Sreeramareddy, V. Srinivasan, J. D. Stanaway, V. Stathopoulou, N. Steel, D. J. Stein, M. A. Stokes, L. Sturua, M. B. Sufiyan, R. A. Suliankatchi, B. F. Sunguya, P. J. Sur, B. L. Sykes, P.N. Sylaja, R. Tabarés-Seisdedos, S. K. Tadakamadla, A. H. Tadesse, G. R. Taffere, N. Tandon, A. T. Tariku, N. Taveira, A. Tehrani-Banihashemi, G. Temam Shifa, M.H. Temsah, A. S. Terkawi, A. G. Tesema, D. J. Tesfaye, B. Tessema, J.S. Thakur, N. Thomas, M. J. Thompson, T. Tillmann, Q. G. To, R. Tobe-Gai, M. Tonelli, R. Topor-Madry, F. Topouzis, A. Torre, M. Tortajada, B. X. Tran, K. B. Tran, A. Tripathi, S. P. Tripathy, C. Troeger, T. Truelsen, D. Tsoi, L. Tudor Car, K. B. Tuem, S. Tyrovolas, U. S. Uchendu, K. N. Ukwaja, I. Ullah, R. Updike, O. A.

Uthman, B. S. C. Uzochukwu, P. R. Valdez, J. F. M. van Boven, S. Varughese, T. Vasankari, F. S. Violante, S. K. Vladimirov, V. V. Vlassov, S. E. Vollset, T. Vos, F. Wagnew, Y. Waheed, M. T. Wallin, J. L. Walson, Y. Wang, Y.P. Wang, M. M. Wassie, M. R. Weaver, E. Weiderpass, R. G. Weintraub, J. Weiss, K. G. Weldegwergs, A. Werdecker, T. E. West, R. Westerman, R. G. White, H. A. Whiteford, J. Widecka, A. S. Winkler, C. S. Wiysonge, C. D.A. Wolfe, Y. A. Wondimkun, A. Workicho, G. M. A. Wyper, D. Xavier, G. Xu, L. L. Yan, Y. Yano, M. Yaseri, N. B. Yimer, P. Yin, P. Yip, B. D. Yirsaw, N. Yonemoto, G. Yonga, S.J. Yoon, M. Yotebieng, M. Z. Younis, C. Yu, V. Zadnik, Z. Zaidi, M. E. S. Zaki, S. B. Zaman, M. Zamani, Z. M. Zenebe, M. Zhou, J. Zhu, S. R. M. Zimsen, B. Zipkin, S. Zodpey, L. J. Zuhlke, C. J. L. Murray, R. Lozano, Measuring performance on the Healthcare Access and Quality Index for 195 countries and territories and selected subnational locations: A systematic analysis from the Global Burden of Disease Study 2016. *Lancet* **391**, 2236–2271 (2018).

53. R Core Team, *R: A Language and Environment for Statistical Computing* (R Foundation for Statistical Computing, 2018); https://www.R-project.org.
54. J. E. Staples, M. Diallo, K. B. Janusz, C. Manengu, R. F. Lewis, W. Perea, S. Yactayo, A. A. Sall; RCA Risk Assessment Team, Yellow fever risk assessment in the Central African Republic. *Trans. R. Soc. Trop. Med. Hyg.* **108**, 608–615 (2014).
55. T. F. Tsai, J. S. Lazuick, R. W. Ngah, P. C. Mafiamba, G. Quincke, T. P. Monath, Investigation of a possible yellow fever epidemic and serosurvey for flavivirus infections in northern Cameroon, 1984. *Bull. World Health Organ.* **65**, 855–860 (1987).
56. M. H. Kuniholm, N. D. Wolfe, C. Y.-H. Huang, E. Mpoudi-Ngole, U. Tamoufe, M. LeBreton, D. S. Burke, D. J. Gubler, Seroprevalence and distribution of Flaviviridae, Togaviridae, and Bunyaviridae arboviral infections in rural Cameroonian adults. *Am. J. Trop. Med. Hyg.* **74**, 1078–1083 (2006).
57. M. Mengesha Tsegaye, B. Beyene, W. Ayele, A. Abebe, I. Tareke, A. Sall, S. Yactayo, M. E. Shibeshi, E. Staples, D. Belay, A. Lilay, A. Alemu, E. Alemu, A. Kume, A. H/Mariam, O. Ronveaux, M. Tefera, W. Kassa, A. Bekele Weyessa, D. Jima, A. Kebede, A. Tayachew, Sero-prevalence of yellow fever and related Flavi viruses in Ethiopia: A public health perspective. *BMC Public Health* **18**, 1011 (2018).
58. A. O. Kwallah, S. Inoue, A. W. Thairu-Muigai, N. Kuttoh, K. Morita, M. Mwau, Seroprevalence of yellow fever virus in selected health facilities in Western Kenya from 2010 to 2012. *Jpn. J. Infect. Dis.* **68**, 230–234 (2015).

59. S. A. Omilabu, J. O. Adejumo, O. D. Olaleye, A. H. Fagbami, S. S. Baba, Yellow fever haemagglutination-inhibiting, neutralising and IgM antibodies in vaccinated and unvaccinated residents of Ibadan, Nigeria. *Comp. Immunol. Microbiol. Infect. Dis.* **13**, 95–100 (1990).
60. M. Baba, C. H. Logue, B. Oderinde, H. Abdulmaleek, J. Williams, J. Lewis, T. R. Laws, R. Hewson, A. Marcello, P. D'Agaro, Evidence of arbovirus co-infection in suspected febrile malaria and typhoid patients in Nigeria. *J. Infect. Dev. Ctries.* **7**, 51–59 (2013).
61. E. C. Farnon, L. H. Gould, K. S. Griffith, M. S. Osman, A. E. Kholy, M.-E. Brair, A. J. Panella, O. Kosoy, J. J. Laven, M. S. Godsey, W. Perea, E. B. Hayes, Household-based sero-epidemiologic survey after a yellow fever epidemic, Sudan, 2005. *Am. J. Trop. Med. Hyg.* **82**, 1146–1152 (2010).
62. World Health Organization, The Weekly Epidemiological Record (WER) (2019); http://www.who.int/wer/en/.
63. World Health Organization, Disease Outbreak News (DONs) (2020); http://www.who.int/csr/don/en/.
64. Disease burden; https://www.who.int/teams/immunization-vaccines-and-biologicals/immunization-analysis-and-insights/surveillance/monitoring.
65. F. Hartig, F. Minunno, S. Paul, D. Cameron, T. Ott, Bayesiantools: General-purpose MCMC and SMC samplers and tools for Bayesian statistics, R package version. R package version 0.1.3 (2017).
66. N. Thompson Hobbs, M. B. Hooten, *Bayesian Models: A Statistical Primer for Ecologists* (Princeton Univ. Press, 2015).
67. S. N. Wood, *Generalized Additive Models: An Introduction with R* (Chapman and Hall/CRC, 2017).
68. A. Liaw, M. Wiener, Classification and regression by randomForest. *R News* **2**, 18–22 (2002).
69. G. Ridgeway, B. Greenwell, B. Boehmke, J. Cunningham, GBM Developers, Generalized boosted regression models. R package version. 1, 55 (2006).
70. C. Forbes, M. Evans, N. Hastings, B. Peacock, *Statistical Distributions* (John Wiley & Sons, 2011).
71. N. A. Weiss, *A Course in Probability* (Addison-Wesley, 2006).
72. C. G. Bowsher, P. S. Swain, Identifying sources of variation and the flow of information in biochemical networks. *Proc. Natl. Acad. Sci. U.S.A.* **109**, E1320–E1328 (2012).
